# Immunization With Bovine Milk Casein Results in Enteric Nervous System Pathology in a Mouse Model of Neuroinflammation

**DOI:** 10.1111/nmo.70142

**Published:** 2025-08-14

**Authors:** Rittika Chunder, Alicia Weier, Young An, Angelika Zoons, Maik Hintze, Stefanie Kuerten

**Affiliations:** ^1^ Institute of Neuroanatomy, Medical Faculty University of Bonn and University Hospital Bonn Bonn Germany; ^2^ Department of Microbiology and Immunology, Peter Doherty Institute for Infection and Immunity University of Melbourne Melbourne Victoria Australia

**Keywords:** casein, cross‐reactivity, enteric nervous system, inflammation, milk

## Abstract

**Background:**

Multiple sclerosis (MS) is an immune‐mediated demyelinating disease of the central nervous system (CNS) with evidence of autoimmune attack also on the enteric nervous system (ENS). The role of different dietary antigens, including bovine milk proteins, in the exacerbation of MS symptoms has previously been discussed.

**Methods:**

In a mouse model of neuroinflammation, we characterized the extent of ENS pathology in animals that were immunized with different bovine milk antigens using electron microscopy, immunohistochemistry, molecular biology, and cell culture as key methods.

**Results:**

Our data demonstrate that immunization of mice with bovine milk casein resulted in ENS pathology, which is in line with our previous findings where casein‐immunized mice also exhibited demyelination in the CNS. Furthermore, development of ENS pathology was most likely due to a combination of cellular and humoral factors, as confirmed by our observation of CD3^+^ T cell infiltration in the *tunica muscularis* and binding of serum antibodies from casein‐immunized mice to glial cells in the myenteric plexus.

**Conclusion:**

The findings presented in this paper reflect that exposure to bovine casein can result in axolysis in the myenteric plexus possibly as a result of molecular mimicry and antibody cross‐reactivity between casein and antigen(s) expressed by the ENS.

## Graphical abstract

Experimental study design. Mice were immunized with either α‐Lactalbumin or casein, and the resulting pathology of the enteric nervous system (ENS) was assessed using electron microscopy and immunofluorescence imaging. In vitro culture of isolated cells of the ENS was used to complement the in vivo findings.

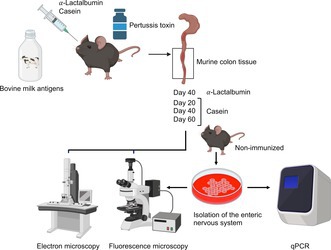

**Summary**

- Exposure to bovine milk antigens, in particular casein, is shown to result in the development of enteric nervous system pathology in a mouse model of neuroinflammation.
- Mice immunized with bovine milk casein not only displayed axolysis of the myenteric plexus but serum from the same mice recognized enteric glial cell antigens in vitro.
- Our finding is of particular importance in patients with neuroinflammatory disorders like multiple sclerosis where an intricate tripartite interaction between dietary antigens, the enteric nervous system and the central nervous system has been suggested.

## 1 Introduction

Multiple sclerosis (MS) is a neuroinflammatory disease, traditionally considered to be central nervous system (CNS)‐specific [1]. However, recent studies have established a mechanistic link between an autoimmune attack on the CNS and the development of enteric nervous system (ENS) pathology in patients with MS [2, 3], thus challenging our understanding of the immunopathogenesis of this disorder.

Clinically, it has been reported that approximately 65% of patients, independent of the type of MS, display gastrointestinal (GI) dysfunction with symptoms including dysphagia, dyspepsia, and fecal incontinence [4]. According to one study, a third from a cohort of 385 patients with MS reported GI symptoms prior to a demyelinating event in the CNS, which is typically associated with the disease [5]. Indeed, GI symptoms like decreased peristalsis can result from lesions in the brainstem [6] and spinal cord [7]; however, alterations in the ENS, including degeneration or functional impairment of enteric neurons and/or glial cells and interstitial cells of Cajal, can also manifest as abnormalities in the motor functions of the GI system [8, 9, 10]. Histopathologically speaking, in addition to the presence of inflammatory lesions in the brain and spinal cord [11], bowel resectates of the myenteric plexus from patients with MS showed loss of enteric nerve fibers and enterogliosis [2]. While it is recognized that the ENS can be a target of autoimmune attacks in MS [2], the precise molecular and cellular components associated with and resulting in ENS pathology remain to be elucidated.

Given that loss of immunological self‐tolerance in MS has been attributed to an intricate interplay between genetic and environmental factors [12, 13], it is tempting to speculate that GI symptoms in patients may result from damage to the ENS initiated by loss of tolerance to otherwise harmless food antigens. Although the precise mechanisms leading to loss of (oral) tolerance remain to be clearly deciphered [14], it can result from, for example, molecular mimicry between specific food and self‐antigens triggering in antibody cross‐reactivity between the two [15, 16, 17].

On the one hand, there is evidence of how specific dietary patterns, such as consumption of a high salt diet [18], can result in the development of enteric inflammation, damage to the intestinal barrier, and an increase in the number of cytotoxic T cells [19, 20]. On the other hand, not only has a positive association between consumption of bovine milk and the prevalence of MS [21, 22, 23] been proposed, but our group has previously demonstrated antibody cross‐reactivity between bovine casein and myelin‐associated glycoprotein (MAG), possibly as a result of molecular mimicry between the two antigens [24]. Breakdown of oral tolerance to casein in patients with MS can be triggered by disturbances in antigen uptake and presentation of the immunogenic epitopes of specific food antigens [25, 26]. Whether antibodies to bovine milk antigens, for example, casein, have the ability to induce ENS pathology by similar cross‐reactivity mechanisms remains unknown.

Using diet and autoimmunity as two contributors of disease pathogenesis, in the present study, we investigated the development of ENS pathology in a mouse model of neuroinflammation. To this end, we used intestinal tissue of mice immunized with bovine milk casein to check for the development of GI tract pathology. In line with our previous observation where we demonstrated pathogenic binding of serum antibodies from casein‐immunized mice to CNS tissue [24], here we show a similar negative effect of bovine milk casein on the ENS.

## 2 Materials and Methods

### 2.1 Mice and Immunizations

The mice used in this study were from the same cohort reported by Chunder et al. [24]. Briefly, *N* = 25 eight‐week old female wild‐type (WT) C57BL/6J (B6) mice (Charles River) were kept under specific pathogen‐free conditions at the animal facility of the Franz‐Penzoldt‐Zentrum, Erlangen, Germany (approval by the Regierung von Unterfranken, RUF‐55.2.2‐2532‐2‐575‐5). Specific pathogen‐free conditions included the absence of, but were not limited to, mouse hepatitis virus, Theiler's murine encephalomyelitis virus, mouse cytomegalovirus, lymphocytic choriomeningitis virus, *Helicobacter* spp., *Streptococcus* spp., *Pasteurella* spp., *Klebsiella* spp., helminths, ectoparasites and enteric pathogenic protozoa. All animal experiments were performed in accordance with the German Law on the Protection of Animals and the “Principles of laboratory animal care” [27]. Animal experiments also complied with the ARRIVE (Animal Research: Reporting of In Vivo Experiments) guidelines [28].

A total of four cohorts of B6 mice were immunized as follows: Three cohorts of *N* = 5 mice/cohort were subcutaneously (s. c.) immunized with 200 μg of casein solution from bovine milk (Sigma Aldrich; Cat. No. C4765) and one cohort of *N* = 5 mice/cohort was immunized s.c. with 200 μg of bovine milk α‐Lactalbumin (Sigma‐Aldrich; Cat. No. L6010). α‐Lactalbumin was included as a representative bovine milk non‐casein whey protein antigen.

The different antigens were emulsified (1:1) in complete Freund's adjuvant (CFA) containing 5 mg/mL *Mycobacterium tuberculosis* H37Ra (BD Difco; Cat. No. BD231141) prior to s.c. injections; additionally, 200 ng pertussis toxin (List Biological Laboratories; Cat. No. #180; LOT number 180243A1) was delivered intraperitoneally on the day of immunization and 48 h later. One cohort each of the casein‐immunized mice was sacrificed on Days 20, 40, and 60. Mice immunized with α‐Lactalbumin were sacrificed on Day 40. *N* = 5 mice remained as non‐immunized controls.

To harvest bovine milk casein‐specific serum immunoglobulin (Ig), a second group of *N* = 6 female WT B6 mice (Charles River) was used in this study. Animals were kept under specific pathogen‐free conditions in the animal facility of the House for Experimental Therapy (HET 4) of the University Hospital Bonn (approved by the Landesamt für Natur, Umwelt und Verbraucherschutz (LANUV) NRW, file number 81‐02.04.2021.A146). The mice were immunized as described above with casein solution from bovine milk and were sacrificed on Day 40 post immunization. An additional cohort of non‐immunized mice (*N* = 12) was included.

### 2.2 Preparation of Tissue Sections for Electron Microscopy

Mice were sacrificed by CO_2_ and perfused with 4% paraformaldehyde (PFA) in phosphate‐buffered saline (PBS) (pH 7.4). The entire intestine from the distal to the proximal end was dissected. Fecal matter was removed, and the intestine was cut into two pieces: [1] a region containing the jejunum/ileum and [2] colon, of which 0.5 cm of both regions of the intestine were processed for electron microscopy.

Tissue preparation for subsequent electron microscopic analysis of the colon was done as previously described [29]. The tissue pieces were rinsed out using PBS before fixing them in 4% glutaraldehyde/4% PFA/0.002% picric acid in 0.1 M cacodylate buffer (pH 7.2), overnight (O/N) at 4°C. The specimens were rinsed in phosphate buffer and post‐fixed in 1% osmium tetroxide and 1.5% potassium ferricyanide for 4 h at 4°C. Next, the tissues were rinsed again in phosphate buffer for 2 h and dehydrated in an ascending ethanol series. The samples were subsequently penetrated with an ethanol/acetone mixture, pure acetone, an acetone/EPON 812 substitute mixture for 30 min each, and pure EPON 812 substitute, O/N. After the addition of 2% glycidether accelerator DMP‐30 (Carl Roth; Cat. No. 8621.1) to the final volume of the EPON 812 substitute, tissue samples were placed in resin‐filled BEEM capsules and polymerized at 60°C and 80°C. Single silver ultrathin sections were cut, mounted on single Pioloform‐coated copper slot grids (Plano) and stained with lead citrate as well as uranyl acetate. Ultrathin sections of 50 nm were examined with a Zeiss EM 906 transmission electron microscope (Carl Zeiss NTS GmbH) at a cathode voltage of 60 kV.

### 2.3 Electron Microscopic Assessment

Images at the electron microscope were taken as previously described by our group [24, 30]. Briefly, 10 images of the myenteric plexus from both the colon and the jejunum per section were taken at 10,000× magnification. This resulted in a total of 25–30 images per mouse. Five of these images from each section (i.e., *N* = 10–15 images per mouse) were randomly chosen for image analysis. The area of the myenteric plexus (μm^2^), the total number of axons, and the number of damaged axons were quantified using Image J software (version 1.54 k; National Institutes of Health (NIH)).

### 2.4 Immunohistochemistry

Tissues were fixed with 4% PFA by intracardial perfusion as described above. A piece of colon tissue that was 0.5 cm long was immersion‐fixed in 4% PFA and subsequently processed for paraffin embedding. 5 μm thick serial sections of the murine intestinal tissue were cut up to a total of 30 sections per mouse. To process the samples for immunohistochemical staining, tissue sections were deparaffinized with xylene and rehydrated using a descending ethanol series from 100% to 70%. Heat‐mediated antigen retrieval was performed using 0.1 M sodium citrate (pH 6.0) for 10 min. Sections were blocked in 5% milk powder in tris‐buffered saline (TBS) with 0.05% Tween‐20 (TBS‐T) at room temperature (RT) for 1 h and then incubated with primary antibodies diluted in 0.5% milk powder in TBS‐T either at RT for 3 h or O/N at 4°C. For the detection of T cells, B cells, and macrophages, colon tissue was incubated with anti‐CD3 (Abcam; Cat. No. ab135372; dilution 1:150), anti‐CD45R (Thermo Fisher; Cat. No. 14‐0452‐82; dilution 1:200) and anti‐CD11b (Thermo Fisher; Cat No. 14‐0112‐82; dilution 1:100) antibodies, respectively.

Tissue sections were subsequently incubated with the corresponding secondary antibodies for 1–2 h at RT. For the localization of murine macrophages, T cells, or B cells, a donkey anti‐mouse Cy3 (Jackson ImmunoResearch; Cat. No. 715‐165‐151; dilution 1:400), donkey anti‐rabbit Cy3 (Jackson ImmunoResearch; Cat. No. 711‐165‐152; dilution 1:400) or a donkey anti‐rat Cy2 (Jackson ImmunoResearch; Cat. No. 712‐225‐153; dilution 1:400) secondary antibody, respectively, was applied. All secondary antibodies were diluted in TBS‐T.

For double stains, sections were washed with TBS‐T and incubated with the second primary antibody at RT for 3 h. The corresponding secondary antibodies were diluted as mentioned above and incubated at RT for 1 h. All sections were washed with TBS‐T and mounted with Fluoroschield mounting medium containing 4′,6‐diamidino‐2‐phenylindole (DAPI) (Abcam; Cat. No. ab104139). Every staining contained a secondary antibody‐only control, and a section of the murine spleen was used as a positive control for the CD3, B220, and CD11b staining.

Images were acquired using either a Leica DMi8 inverted microscope equipped with the Leica LASX Thunder software or an A1‐HD25 inverted confocal microscope (Nikon, Japan) and an A1 large‐field‐of‐view camera (Nikon, Japan). Quantification of images was done using Image J (version 1.54 k; NIH).

### 2.5 Antigen‐Specific Antibody Binding

Colon tissue from non‐immunized WT mice was deparaffinized as mentioned above. Heat‐mediated antigen retrieval was performed using 0.1 M sodium citrate (pH 6.0) for 10 min. For the identification of a specific antigen–antibody binding pattern on murine gut tissue using serum from either mice immunized with casein or α‐Lactalbumin from bovine milk or non‐immunized mice, a Mouse‐on‐Mouse (M.O.M) immunodetection kit (Vector Laboratories; Cat. No. BMK‐2202) was used. Tissue sections were treated as per the manufacturer's instructions mentioned in the M.O.M immunodetection kit. Dilutions (1:10) of serum from the differently immunized mice were used as primary antibody. Streptavidin conjugated to Alexa Fluor 488 (Thermo Fisher; Cat. No. S32354) was used following incubation with M.O.M biotinylated anti‐mouse IgG reagent (provided in the kit).

A sequential double staining with anti‐glial fibrillary acidic protein (GFAP) antibody (Abcam; Cat. No. ab4674; dilution 1:500) for the visualization of enteric glial cells was performed before the sections were counter‐stained with DAPI, coverslipped, and images were acquired as mentioned above.

### 2.6 Primary Mouse ENS Culture

Primary ENS cells were isolated and cultured as previously described by Weier et al. [30]. For each culture, one adult mouse was used. After perfusion using Hanks' balanced salt solution (HBSS), the intestine was cut into four individual pieces, and fecal matter was removed. The longitudinal muscular layer with the attached myenteric plexus (LMMP) was dissected out, and the isolated LMMP sections were transferred into a gentleMACS C Tube (Miltenyi Biotec; Cat. No. 130‐093‐237) containing HBSS supplemented with fetal bovine serum (FBS), type II collagenase, and bovine serum albumin (BSA). Chemical/mechanical digestion was done using the gentleMACS Octo Dissociator with heaters (Miltenyi Biotec). Cells were centrifuged, and the pellet was resuspended in HBSS containing trypsin and subjected to a second digestion step using the gentleMACS Octo Dissociator. The cells were passed through a 70‐μm cell strainer and finally resuspended in Neurobasal A medium (Thermo Fisher; Cat. No. 10888022) containing 1% B‐27 (Thermo Fisher; Cat. No. 17504044), 1% FBS, 1% L‐glutamine (Thermo Fisher; Cat. No. 25030081), 1% penicillin–streptomycin, and 10 ng/mL glial‐derived neurotrophic factor (Thermo Fisher; Cat. No. PHC7054) as supplements. Cells were seeded onto a 24‐well plate coated with poly‐D‐lysine and mouse laminin (Thermo Fisher) and incubated at 37°C and 5% CO_2_ for 9–10 days. Characterization of the ENS cells has been described elsewhere [30].

### 2.7 Immunocytochemistry

ENS cells were cultured for 9 days, washed with pre‐warmed PBS, and then fixed with 4% PFA in PBS for 10 min. Cells were subsequently permeabilized with 0.1% Triton X‐100 in PBS at RT for 20 min. Blocking was done with 5% donkey serum in PBS (with 0.05% Tween‐20) at RT for 1 h. Cells were incubated at RT for 2 h with purified (total) IgG from pooled sera of *N* = 3–4 casein‐immunized mice diluted 1:10 in TBS‐T. Total immunoglobulin G was purified from the sera using the Mouse Antibody Purification Kit (Abcam; Cat. No. ab128745) following the manufacturer's instructions. IgG from non‐immunized mice served as a control antibody.

A biotinylated goat anti‐mouse IgG antibody (Abcam; Cat. No. ab6788) was used as a secondary antibody. Cells were then incubated in the dark with Alexa Fluor 647 coupled to streptavidin at RT for 45 min. For double staining, anti‐GFAP and anti‐proteolipid protein (PLP) (Abcam; Cat. No. ab254363; dilution 1:50) antibodies as representative markers for enteric glial cells were used. Cells were incubated with the corresponding secondary antibodies, counter‐stained with DAPI, and imaged as described above. Each staining also contained a negative control without a primary antibody.

### 2.8 RNA Extraction and Quantitative PCR

Isolation of RNA from mouse ENS culture was done using the RNeasy Mini kit (Qiagen; Cat. No. 74104) as per the manufacturer's guidelines. RNA from murine mammary gland tissue (Zyagen; Cat. No. MR‐414‐L1‐C57) was used as a positive control for the detection of casein genes. The RNA was reverse transcribed to complementary DNA (cDNA) using the High‐capacity cDNA Reverse Transcription Kit (Applied Biosystems; Cat. No. 4368814) according to the manufacturer's instructions. cDNA was used as a template for subsequent end‐point and qPCR analyses. *Actb* was used as a housekeeping gene control. Relative quantification of mRNA levels was performed using a Roche LightCycler 480 instrument. The qPCR runs included a no‐template control for every primer set; data were analyzed using the ΔΔcycle threshold (C_t_) method.

### 2.9 Statistical Analysis

For statistical analysis, GraphPad Prism (version 10; GraphPad Software Inc.) was used. A Shapiro–Wilk normality test was used to confirm whether the data set followed a normal distribution, and QQ plots were generated for verification. Accordingly, differences between two parametric groups were assessed using either a *t*‐test or one‐way ANOVA, while for non‐parametric data sets, a Mann–Whitney *U*‐test was used. For both parametric and nonparametric datasets, a significance level of 5% was chosen. The statistical tests used for the different analyses are provided in the figure legends. Only significant differences are indicated in the figures.

## 3 Results

### 3.1 Mice Immunized With Casein Exhibit ENS Pathology

In a previously published study [24], we had shown that the CNS is a target of autoimmune insult specifically in mice that were immunized with bovine milk casein. Here we quantified the extent of ENS pathology in the same cohorts of mice. Briefly, mice immunized with casein solution from bovine milk were sacrificed at three different time points (i.e., Days 20, 40 and 60) after immunization (*N* = 5 in each cohort). Mice that had been immunized with α‐Lactalbumin were killed on Day 40 and were included as a control group. All cohorts were observed daily for the development of any clinical signs [24].

In addition to the weakness and spasticity of the limbs as clinical symptoms that were exhibited by the casein‐immunized group of mice as described by us [24], some of the casein‐immunized animals, in particular, *N* = 3/5 mice at Day 40 and *N* = 2/5 mice at Day 60, had difficulties with defecation and had developed what appeared to be meteorism. Difficulties with defecation were assessed by post‐mortem hardening of the abdomen and filling of the terminal colon with a high number of fecal pellets. Given that both these symptoms could indicate gastrointestinal dysfunction, myenteric plexus‐containing regions of both the large and small intestine were processed for electron microscopy.

**FIGURE 1:**
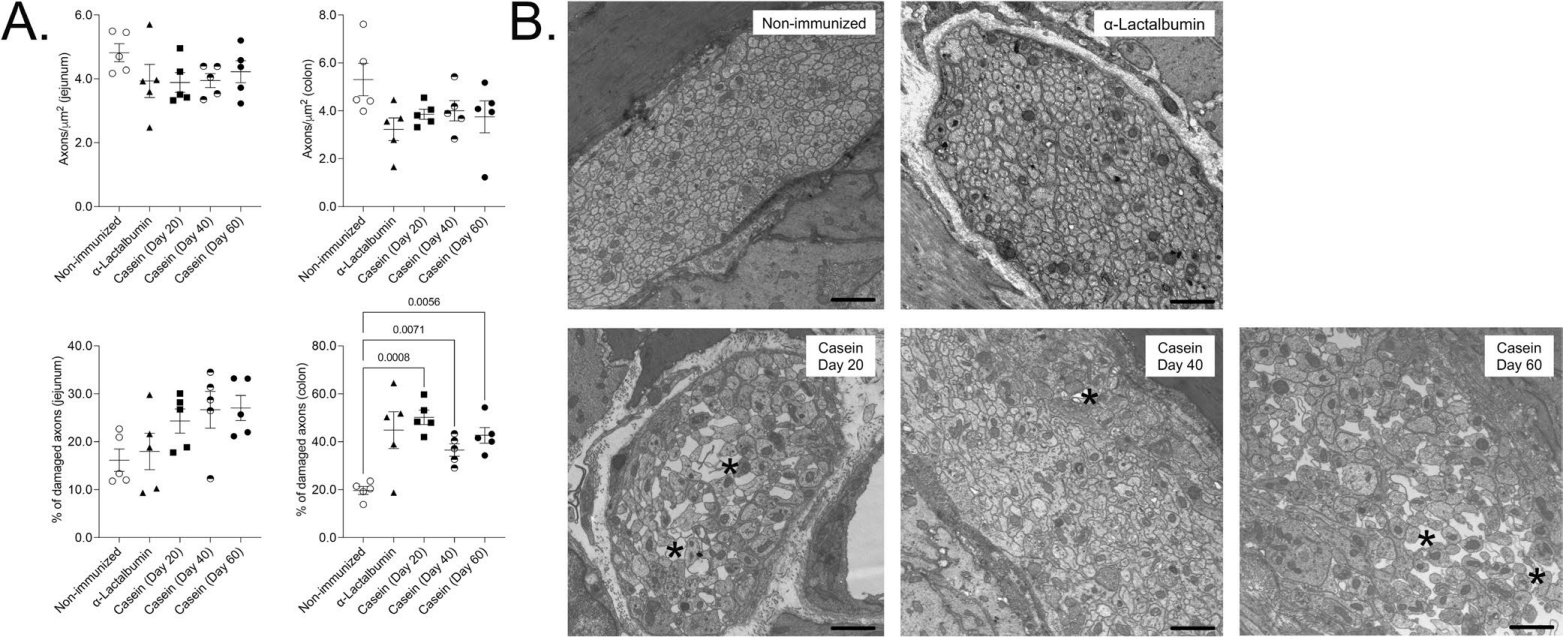
Enteric nervous system pathology in casein‐immunized mice. (A) Total number of axons per μm^2^ of surface area of the myenteric plexus and axonal pathology in the different cohorts of mice were quantified. Each data point represents the mean ± standard error of the mean (SEM) for each mouse. (B) Representative electron microscopic (EM) images of the myenteric plexus of the large intestine of casein‐immunized mice sacrificed on three different time points, that is, Days 20, 40, and 60 post‐immunization. EM images from α‐Lactalbumin‐immunized and non‐immunized mice as two control cohorts are shown in addition. Exemplary axolytic or damaged axon is marked with a star (*). The scale bars indicate 2 μm.

To quantify the effect of casein‐specific immunity on axonal degeneration in the ENS, the total number of axons/μm^2^ of the myenteric plexus of both the jejunum and colon was analyzed. As shown in Figure 1A, no significant differences in the total number of axons/μm^2^ were observed neither between the different groups nor between the time points of sacrifice of the casein‐immunized cohorts, independent of the region of the intestine.

In addition, axons that were in a state of degeneration at the time point of sacrifice in the differently immunized mouse cohorts were quantified as axolytic. While no differences between the different groups were observed in the jejunum, the percentage of damaged axons in the colon, on average, was highest in the mice that were sacrificed at time point Day 20 post‐immunization compared to the non‐immunized cohort. There was no statistical significance in the percentage of damaged axons observed between the α‐Lactalbumin‐immunized and non‐immunized mice.

### 3.2 ENS‐Infiltrating T Cells Are Present in the *Tunica Muscularis* of Casein‐Immunized Mice

On the one hand, using electron microscopy we observed no significant differences in the percentage of damaged axons in the jejunum between the different cohorts. On the other hand, it has been reported that the myenteric plexus is more prominent in the colon [31] than in areas of the small intestine; therefore, directing our focus primarily to the large intestine.

To investigate the mechanism behind the observed ENS pathology, colon tissue was stained for the detection of infiltrating T cells, B cells, and macrophages using CD3, B220, and CD11b as markers, respectively. α‐Lactalbumin‐ (*N* = 5), casein‐ (*N* = 5/time point of sacrifice) and non‐immunized (*N* = 3) mice were used for the fluorescence staining. 7–10 sections from a total of 30 serially acquired sections were randomly selected from every mouse of the differently immunized groups and stained for the different markers. Quantification of cellular infiltration was done using these stained sections. Colon tissue was imaged as a whole, and DAPI^+^ CD3^+^ cells found only between the two muscle layers containing the myenteric plexus (*tunica muscularis*) were included in the analysis.

**FIGURE 2:**
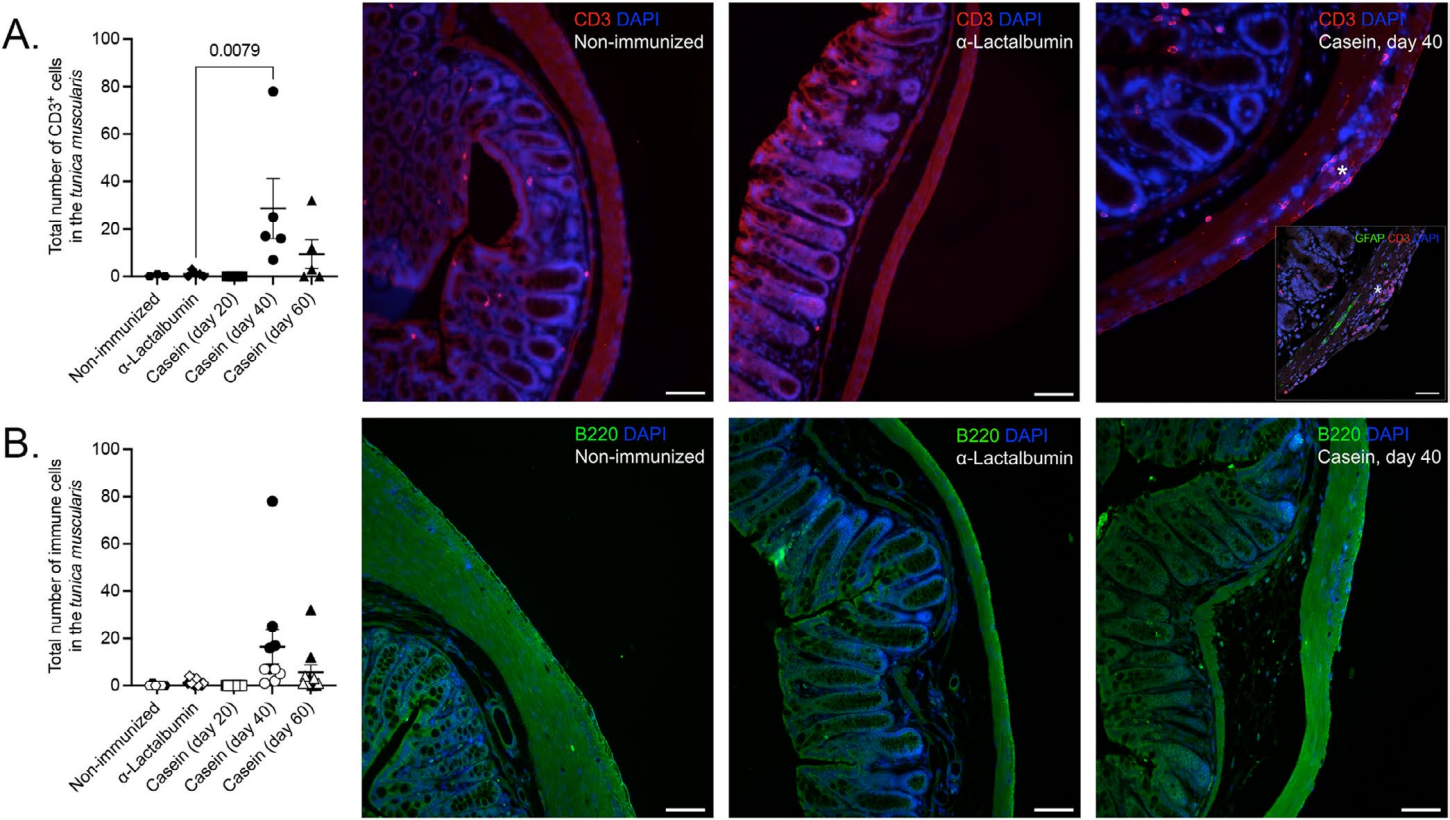
Inflammatory infiltrates in the enteric nervous system of casein‐immunized mice. Paraffin‐embedded sections of the colon were stained with (A) CD3 to detect T cells and with (B) B220 to detect B cells in mice that were immunized with the casein and sacrificed at the different time points vs. α‐Lactalbumin‐immunized and non‐immunized mice. The total number of *tunica muscularis*‐infiltrating CD3^+^ cells only (A) and the total number of CD3^+^ (black symbols) and B220^+^ cells (white symbols) (B) for every mouse is shown. For the quantification of the density of CD3^+^ cells in the *tunica muscularis*, Mann–Whitney *U* test was used. Clusters of CD3^+^ T cells in the *tunica muscularis* from a representative image of the casein‐ immunized cohort are indicated with a star (*). The insert represents the spatial relationship between GFAP^+^ myenteric plexus and CD3^+^ cells. Scale bars: 50 μm. DAPI, 4′,6‐diamidino‐2‐phenylindole; GFAP, glial fibrillary acidic protein.

Mice in the casein‐immunized cohort that were sacrificed on Day 40 had the highest density of CD3^+^ cells in the *tunica muscularis* compared to the other groups, both in terms of the total number of cells/mouse and also the average number of cells/section/mouse (Figure 2A). Mice that were immunized with α‐Lactalbumin (*N* = 5) had < 5 *tunica muscularis*‐infiltrating CD3^+^ T cells in total from all the sections that were analyzed. For the remaining groups, the density of CD3^+^ cells was comparably low, except for one casein‐immunized mouse sacrificed on Day 60, which had a high number of CD3^+^ immune cells (> 30 cells in total) in the regions associated with and surrounding the myenteric plexus (Figure 2A).

The number of B cells in the *tunica muscularis*, on the other hand, did not exceed 3–4 cells/section in all the groups analyzed. Due to the very low numbers of B220^+^ cells in the *tunica muscularis*, no statistical analysis was done. Nevertheless, 2/5 α‐Lactalbumin‐ and 4/5 casein‐immunized mice had at least 3–4 B cells in > 2 sections of the total of 7–10 sections analyzed per mouse (Figure 2B). The total number of CD3^+^ and B220^+^ immune cells in the *tunica muscularis* of the differently immunized groups of mice is summarized in Figure 2B.

No CD11b^+^ cells were detected in the *tunica muscularis* in any of the cohorts.

### 3.3 Serum Igs From Casein‐Immunized Mice Bind Primarily to GFAP ^+^ Cells in the Myenteric Plexus of Non‐Immunized Mice

Serum from α‐Lactalbumin‐ (*N* = 5) and casein‐ (*N* = 5) immunized mice sacrificed on Day 40 after antigen injection was incubated on murine colon sections from a non‐immunized mouse. A double staining with GFAP was done to localize the IgG binding in relation to the myenteric plexus. Serum from a cohort of non‐immunized mice was used as a control.

**FIGURE 3:**
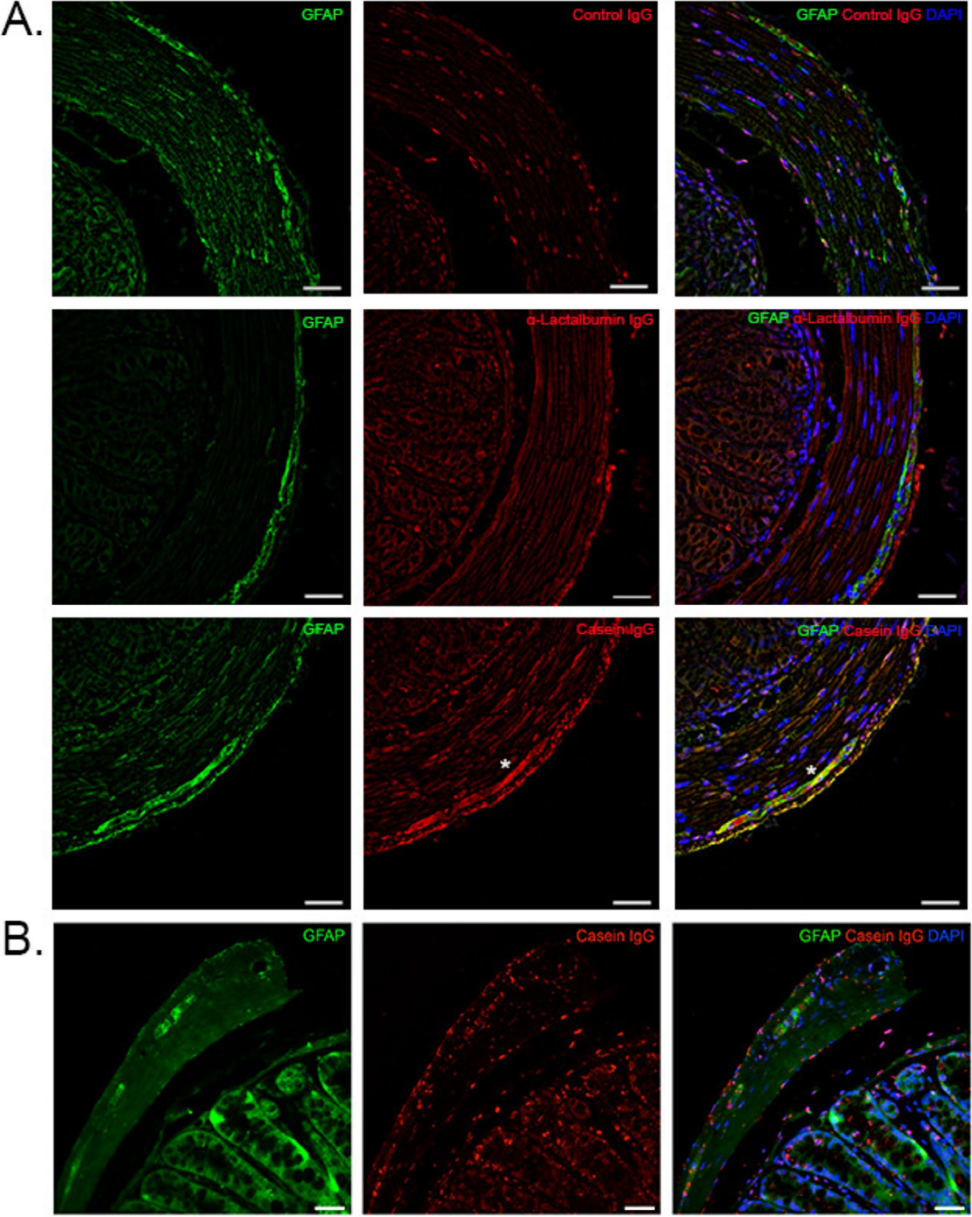
Binding of serum immunoglobulin (Ig)G from casein‐immunized mice to colon sections of healthy mice. (A) Incubation of serum IgG from casein‐immunized, α‐Lactalbumin‐immunized and non‐immunized mice on murine colon sections containing the myenteric plexus, counterstained for glial fibriallary acidic protein (GFAP)^+^ enteric glial cells. Scale bars: 50 μm. (B) Colocalization of GFAP and anti‐casein sera on colonic sections from non‐immunized/healthy mice. Scale bars: 10 μm. Areas of anti‐casein antibody and GFAP colocalization from representative images of the casein immunized cohort is indicated with a star (*) (A and B). DAPI, 4′,6‐diamidino‐2‐phenylindole.

Four of five serum samples from the casein‐immunized cohort showed strong binding to the myenteric plexus. Furthermore, the cytoplasmic binding of serum Igs from mice that were immunized with casein co‐localized with GFAP^+^ cells (Figure 3A). One of five sera from α‐Lactalbumin‐immunized mice presented similar co‐localization results, although the staining pattern was more nuclear than cytoplasmic (Figure 3A). None of the sera from non‐immunized mice presented co‐localization with GFAP^+^ cells.

Co‐staining with GFAP was repeated using sera from a second independent cohort of mice, also sacrificed on Day 40 after immunization. *N* = 6 non‐immunized mice were included as a control. Antibodies from 4/6 casein‐immunized mice (67%) showed cytoplasmic staining patterns of the myenteric plexus (Figure 3B). On the one hand, some cells were double positive for GFAP and casein; on the other hand, others were only either casein IgG‐ or GFAP‐positive, indicating that casein antibodies bind not only to GFAP^+^ enteric glial cells, but also to other GFAP^−^ cell populations in the ENS. Conversely, not all GFAP^+^ enteric glial cells were targeted by serum IgG from casein‐immunized mice (Figure 3B).

### 3.4 Purified IgG From Casein‐Immunized Mice Binds to Primary ENS Cells

Antibodies to bovine casein binding to the myenteric plexus therefore triggered our interest to be potentially pathogenic to cells of the ENS; drawing a parallel to our observation with respect to oligodendrocytes in the CNS [24]. Accordingly, we wanted to further characterize the individual binding pattern of casein‐specific antibodies to murine ENS cells.

**FIGURE 4:**
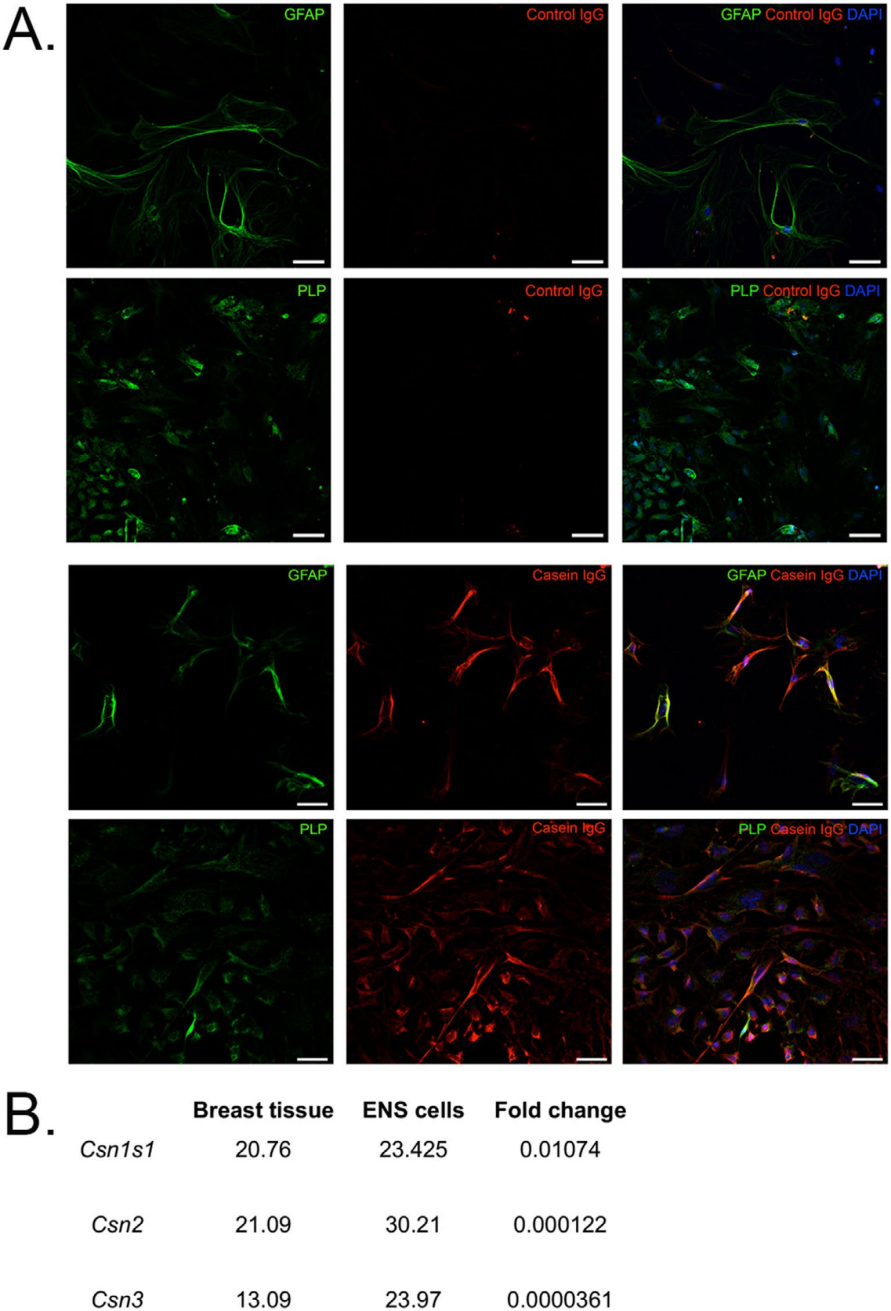
Serum immunoglobulin (Ig)G from immunized mice recognize antigens other than casein expressed by enteric glial cells. (A) Binding of anti‐casein IgG to cells of the enteric nervous system (ENS). Double staining of mixed a culture of ENS cells of non‐immunized mice with an anti‐glial fibrillary acidic protein (GFAP) and anti‐proteolipid protein (PLP) antibodies and either purified IgG from casein‐immunized mice or from non‐immunized mice (control IgG). Scale bars indicate 10 μm. DAPI, 4′,6‐diamidino‐2‐phenylindole. (B) Total RNA was extracted from murine primary ENS culture and relative expression of the different casein genes (*Csn1s1*, *Csn2*, and *Csn3*) was calculated using *Actb* as housekeeping gene.

To this end, immunocytochemistry using purified IgG from casein‐immunized mice was performed on a primary ENS culture. Total IgG purified from *N* = 3–4 casein‐immunized mice was pooled and tested on a primary mixed culture of ENS cells as shown in Figure 4A. When incubated with anti‐casein IgG and either anti‐GFAP antibody or anti‐PLP antibody, where PLP is another enteric glial cell marker [32], a co‐localization of the two fluorochromes on the same ENS cell was observed. Our staining indicated that purified IgG from casein‐immunized mice recognized antigens expressed by GFAP^+^ and PLP^+^ enteric glial cells. To verify that the double staining of GFAP^+^/PLP^+^ ENS cells was specific for serum IgG purified from casein‐immunized mice, the same double staining procedure was repeated with IgG from non‐immunized mice. As shown in Figure 4A, no staining was observed when purified IgG from serum of healthy non‐immunized mice was used.

### 3.5 Murine ENS Cells Do Not Express Casein Genes

Given that sera from casein‐immunized mice showed strong binding to the myenteric plexus of non‐immunized control mice, two possible hypotheses arose: either there was direct binding of anti‐bovine casein IgG to endogenously expressed mouse casein or, as is the case with the myelin antigen MAG, there is molecular mimicry between casein and (other) antigenic structures expressed by the ENS [24]. However, because the ENS does not express MAG [33], we checked the expression of the different casein genes (*Csn1s1*, *Csn2*, and *Csn3* encoding αS1‐, β‐ and κ casein, respectively) by the ENS using RNA isolated from primary cells isolated from non‐immunized murine intestinal tissue. To quantify the relative amount of the expression of different casein genes, qPCR was performed using *Actb* as a housekeeping gene and murine breast tissue as a positive control. The expression level of the different casein genes in the ENS cells was several cycle numbers higher compared to the expression in the breast tissue (Figure 4B), indicating that casein is not expressed by the murine ENS under physiological conditions.

## 4 Discussion

Studies from our laboratory and others [21, 22, 23, 24] have previously highlighted the negative effect of consumption of bovine milk on the aggravation of symptoms in different autoimmune disorders. However, the primary focus of these studies [23, 24] was the influence of an immune response against different bovine milk antigens on CNS demyelination. Indeed, the same dietary antigens have the potential to modulate ENS functions [34, 35], especially given that the ENS runs along the entire GI tract—the site of absorption of ingested nutrients [36]. On the one hand, a healthy gastrointestinal tract can be traced to the correct functioning of the ENS [37]. On the other hand, permanent or even transient alterations in the ENS can result in a number of GI symptoms, including constipation and rectal hyposensitivity, as those reported in approximately one‐third of patients with MS [5]. In line with previous findings [24, 38], in the current study we reiterate the antigenicity of casein in the context of the development of ENS pathology in mice immunized with this milk antigen.

Casein‐immunized mice that were sacrificed on Day 20, that is, the earliest time point, had the highest number of axolytic enteric neurons in the colon among the three different casein‐immunized cohorts (Figure 1). This finding is, however, contrary to our observation in the spinal cord of the same cohort of mice [24], where exacerbation of CNS pathology was observed to be time‐dependent. Interestingly, the time point of maximum axonal pathology (Day 20) and the presence of *tunica muscularis* (i.e., the circular and longitudinal muscle layers containing the myenteric plexus) infiltrating CD3^+^ immune cells (Day 40 and to a lesser extent Day 60) did not coincide in our cohort (Figure 2). We speculate that axonal pathology observed in the casein‐immunized mice that were sacrificed at the earliest time point was primarily mediated by soluble factors, including cytokines, secreted by lamina propria phagocytes rather than a direct effect of immune cell extravasation and migration into the myenteric plexus [39, 40, 41]. As shown in Figure 2, the maximum density of infiltrating CD3^+^ T cells in the *tunica muscularis* was at Day 40 post‐immunization with bovine milk casein, whereas at the last time point (Day 60), most of the T cells had disappeared.

We observed no *tunica muscularis*‐infiltrating innate immune cell population neither at the earliest nor at the latest time point. Furthermore, we detected only very few B cells in the *tunica muscularis* of the casein‐immunized cohort. Taken together, it is possible that antigen processing and subsequent activation of CD3^+^ T cells occur in the lamina propria, licensing them to migrate into the myenteric plexus [42, 43]. In other inflammatory diseases of the gastrointestinal tract, like Crohn's disease, it has been established that certain T cell subsets are activated in the context of a compromised mucosal barrier, thereby driving pathology [44].

In addition to T cell infiltration, 67%–80% of casein‐immunized mice displayed antibody cross‐reactivity to GFAP^+^ enteric glial cells. A discrepancy in the percentage of casein‐immunized mice (80% in cohort 1 vs. 67% in cohort 2) that elicited a cross‐reactive antibody response highlights a heterogeneous immune response to milk antigens in individual mice. Such a variation in the induction of antigen‐specific antibodies even in genetically identical mice has been observed before in a previous study [45].

On the one hand, using primary murine ENS cells, we were able to show the binding of purified IgG from casein‐immunized mice to both PLP and GFAP expressing glial cells. On the other hand, purified IgG from the same mice also bound to a population of cells that was negative for both GFAP and PLP, indicating that it is not the enteric glial cells alone that are targeted by these antibodies. Further analysis using different markers needs to be carried out to carefully dissect the multi‐cellular reactivity of anti‐casein antibodies.

As shown in Figure 4, the lack of mRNA expression of the different casein genes, *Csn1s1*, *Csn2*, and *Csn3*, by ENS cells argues against endogenously expressed caseins as an antigenic target. We, therefore, propose a “cross‐reactive” IgG response in casein‐immunized mice as the etiologic agent of the aggravated pathology. However, precisely which enteric glia or neuronal antigen cross‐reacts with which of the three different casein types currently remains unknown. Similar to our previously published results [24], here we suggest that loss of tolerance to the group of milk protein casein(s), one or several of which share sequence homology with ENS antigens, results in antibody cross‐reactivity between the two. We acknowledge that, in addition to the experiments performed, further studies need to be conducted, including isolation of casein‐specific antibodies from the immunized mice and immunoprecipitation followed by mass spectrometric analysis of the cross‐reactive ENS antigen(s).

Our results demonstrate that immunization of mice with casein from bovine milk results in ENS pathology, which is in line with our previous findings where casein‐immunized mice also exhibited demyelination in the CNS. Development of ENS pathology is most likely due to a combination of cellular and humoral factors, as confirmed by our observation of CD3^+^ T cell infiltration in the *tunica muscularis* and anti‐casein IgG deposition in the myenteric plexus.

To conclude, consumption of bovine milk casein has been associated with the development of a number of peripheral as well as neurological diseases [15, 16, 17]. Among others, one study has reported a correlation between the risk of developing schizophrenia and elevation of antibodies to bovine casein prior to disease onset [46]; while another study established a relationship between the consumption of casein and the degree of atherosclerosis [47]. We extend the repertoire of disease pathogenesis associated with the consumption of bovine casein to the development of ENS pathology as a result of exposure to this milk antigen.

Furthermore, it would be interesting to investigate the possible effects of other bovine milk antigens like β‐Lactoglobulin and bovine serum albumin (BSA) on the ENS. Systematic identification of the bioactive components in bovine milk not limited to casein and removal of such immunogenic antigens from the diet of patients would open up new personalized therapeutic interventions and could be used as a complementary therapeutic intervention in addition to disease‐modifying treatments (DMTs) to control the progression of a particular disease, including MS.

## Author Contributions

R.C., A.W., Y.A., and A.Z. performed experiments, analyzed, and interpreted the data. M.H. was involved in acquiring images. S.K. supervised the study. R.C. and S.K. acquired funding and wrote the manuscript. All authors read and approved the final version of the manuscript.

## Conflicts of Interest

A.W., Y.A., A.Z., and M.H. have no conflicts of interest. R.C. has received funding from the DFG under Germany's Excellence Strategy (EXC2151—390873048) and from Novartis (Oppenheim‐Förderpreis für Multiple Sklerose 2023). S.K. reports funding from Novartis, F. Hoffmann‐La Roche, and Sanofi; and speaker fees and consultancy honoraria from Novartis, F. Hoffmann‐La Roche, Sanofi, and Teva. S.K. is supported by the DFG IRTG 2168 (grant no. 272482170) and the DFG project (460333672 CRC1540 EBM), and she is a member of the Excellence Cluster “ImmunoSensation^2^” (EXC2151—390873048). The remaining authors declare that the research was conducted in the absence of any commercial or financial relationships that could be construed as a potential conflicts of interest.

## Data Availability

The data that support the findings of this study are available from the corresponding author upon reasonable request.
